# Tempests and tales: challenges to the study of sex differences in the brain

**DOI:** 10.1186/2042-6410-2-4

**Published:** 2011-04-28

**Authors:** Margaret M McCarthy, Gregory F Ball

**Affiliations:** 1 Departments of Physiology and Psychiatry, Program in Neuroscience, University of Maryland School of Medicine, 655 W. Baltimore St., Baltimore, MD 21210, USA; 2 Department of Psychological and Brain Sciences, Ames Hall, Johns Hopkins University, Baltimore, MD 21218, USA

## 

The topic of sex differences in brain and behavior continues to garner broad interest and generate considerable controversy. A spate of popular books in the past decade has heralded many of the recent advances in the study of the biological basis of human brain differences in relation to sex and gender. Volumes such as Doreen Kimura's *Sex and Cognition *[1], Simon Baron-Cohen's *The Essential Difference: Men, Women and the Extreme Male Brain *[2], Melissa Hines's *Brain Gender *[3] and Louann Brizendine's *The Female Brain *[4] have reviewed, and in some instances overinterpreted, the current state-of-the-art. This flurry of attention has also generated lightning rods for criticism, as evidenced by the two books reviewed here: Rebecca M Jordan-Young's *Brain Storm: The Flaws in the Science of Sex Differences *[5] and Cordelia Fine's *Delusions of Gender: How Our Minds, Society and Neurosexism Create a Difference *[6]. Both books contain much of merit that we think readers of the *Biology of Sex Differences *will agree with, but both books can be vexing in the ways the scientific study of sex differences in brain and behavior is portrayed and the current state-of-the-art is presented.

Jordan-Young wrote *Brain Storm *after she became interested in the causes of variation in human sexual behavior while engaging in a study of human sexuality related to the acquired immunodeficiency syndrome epidemic. During the course of this work, she was struck by the substantial variation that is exhibited by humans in relation to sexual behavior and the difficulty one can encounter in fitting individuals neatly into categories such as man versus woman or homosexual versus heterosexual. In this context, she was fascinated by the claim that there might be a "male or female" or a "gay versus straight" brain. Her interest was piqued by Simon LeVay's 1991 *Science *paper [7] because it employed a very simple categorization of its subjects into "men, women and gay men." She questioned the value of such a scheme, given the lack of sharp categorical boundaries she had experienced in her own work. When trying to understand the possible causes of correlations between brain structure and human sexual behavior identified by Gorski *et al*. [8], LeVay [7] and Swaab [9], to name a few, she learned about the organizational and activational hypothesis proposed by Phoenix *et al*. in 1959 [10]. The now iconic organizational and activational hypothesis codifies the concept that early hormone exposure permanently organizes the neural substrates which will be activated in a sex-specific manner in adulthood by gonadal steroid hormone production. As most readers of this journal will know, whether this hypothesis can be applied to the human brain remains a matter of controversy and a difficult question to address, given the inability to perform the same types of experiments that have clarified the validity of the hypothesis in other species. Jordan-Young reviews, in a thorough and engaging manner, the challenges and pitfalls of trying to study brain sexual differentiation in humans. This is one of the strongest aspects of the book. Human behavior is complex and often does not exhibit marked or categorical sex differences as have been identified in some cases in other species. Both human sexuality and human cognition require multidimensional behavioral analyses to capture the full range of observed variation. To capture this variation and then relate it to a static, one-time snapshot of a brain structure or a hormone measurement is fraught with difficulties and subject to error. One can only make correlations. However, these correlations are a first step that can set the stage for further study. Moreover, even if one takes the arguably simplistic approach of simply defining every human as male versus female, there are numerous robust and reliable sex differences in the size, shape and neurochemical phenotype of specific brain regions in boys versus girls and men versus women. The challenge is to discern how the observed sex differences were established and what they mean. As noted by Jordan-Young, despite the promise of variants in human reproductive development, such as androgen-insensitivity syndrome and congenital adrenal hyperplasia (CAH) or individual cases (such as the famous "Joan/John" instance of a boy whose sex was reassigned as a girl after his penis was accidently and irreversibly damaged), the direction of the arrow of causation (if there is a causal relationship at all) cannot be specified. Frustratingly, after consideration of the iconic Phoenix *et al*. guinea pig study [10], Jordan-Young gives little attention to the voluminous animal studies conducted in the intervening 50+ years, denying the reader the presentation of overwhelming evidence that correlations between brain and behavior can be the result of early organizational effects of steroid hormones or adult activational effects (or both), or of experiences that occur independently of hormones. Instead of presenting a complex and vibrant field that at times produces messy or inconsistent data, she takes a very humanocentric view and suggests that studies of sex differences lack structure and coherence. Moreover, she ignores critiques that have emerged from within the field [10], implying that there is unanimity among scientists studying sex differences regarding the relative contribution of hormones versus other variables. Nothing could be further from the truth, as anyone subject to peer review can tell you. She also ignores the published provision of an experimental road map that lays out the basic protocols needed to first ascertain whether there is a sex difference in a trait, then to investigate whether hormones are involved and, if so, to distinguish possible organizational effects from activational hormonal effects [11]. Nonetheless, if one considers the broader biological context of studies of sex differences in brain and behavior in humans, Jordan-Young's concerns are well-founded. It is indeed difficult to ascertain at present which factors, biological or environmental, cause the documented sex differences in the human brain, although significant evidence suggests that gonadal hormones are among these factors [12]. If one were just looking for a good species among all vertebrates to try to ascertain basic principles that underlie interrelationships among hormones and brain and sex differences, one would certainly not pick *Homo sapiens*!

Cordelia Fine's penning of *Delusions of Gender *was apparently motivated by her observation of her son's kindergarten teacher reading a book which reportedly stated that males do not have the neural wiring required to connect language and emotion (exactly which book this was is not clear, but a little digging suggests it is likely to be by Brizendine [4]. She reports being sufficiently outraged that she decided to research for herself the studies on which such flimflam was based. Fine is an engaging and clever writer who combines sexist quotes from the Victorian era with modern-day quotes from some of the more offensive recent popular books. The not-so-subtle message, that little has changed for women for hundreds of years, is effectively and powerfully conveyed. Fine goes on to review in exacting and voluminous detail the huge number of studies demonstrating how insidiously gender bias pervades our society and how this in turn leads to gender inequality. Given the overwhelmingly strong case that so much of what distinguishes males and females comes from our decidedly different experiences, Fine's frustration with the unfounded claims for "hardwiring" of any sex difference in the human brain is perfectly understandable (Cordelia, we feel your pain). Unfortunately, though, she opts for throwing neuroscientists under the bus instead of directing her anger where it truly and deservedly belongs.

Fine divides her book into three parts, each of which comprises a collection of short chapters designed to make a specific point revealed by wordplay titles such as "Brain Scams" or "Sex and Premature Speculation." Part I is titled "Half-Changed World - Half-Changed Minds" and should have been printed with a warning label for women that even if you are married to a "rare jewel," like Fine apparently is, you are likely to find yourself angry and resentful that you are doing more of the housework while getting less credit for your better curriculum vitae and earning lower pay for equal or more work. It is still a man's world, and Fine wants to be darn sure we appreciate exactly how much of a man's world it still is. Part III hammers this same concept home with detailed discussions of the overwhelming evidence that the perception of gender, both by others and by oneself, begins even before an individual is self-aware and thereby directs all future choices, from playmates and toys to career and lifestyle. There is no arguing with the point Fine is making: gender is an overwhelmingly salient and pervasive parameter that defines each of us in ways we do not even realize. Her big complaint is that any component of these differences be accepted as "biological." She deftly reveals the level of absurdity of some arguments, pointing out that suggestions of an evolutionary basis for a preference for pink by girls and for blue by boys is ridiculous in the face of the recent emergence of this Western culture color code in only the past 50 years. She is witty and acerbic, issuing lacerating asides and derisive dismissals. She uses favorite punching bags over and over to make her points, with Lawrence Summers, the former president of Harvard University, mentioned so often you would think he had spent his entire career publicly speaking about the inferiority of women. Her irritation is equally great or greater with Simon Baron-Cohen [2] and Louann Brizendine [4], both of whom have written popular books, and who Fine essentially accuses of being charlatans. After the hundredth or so insulting aside, it begins to seem a bit mean-spirited.

But it is in Part II, which she titled "Neurosexism," that Fine really disappoints. Throughout the book she repeatedly warns the reader of the perils of preconceived notions, inherent and hidden biases and seeing only what we want to see. Yet, that is exactly the behavior she there engages in. Her use of the inflammatory and made-up term "neurosexism" is further supported by her use of prejudicial words such as "neurononsense" and "neuroscientific" (the closeness to "pseudoscientific" is undoubtedly intentional). She goes so far as to say, "sexism dressed up in neuroscientific finery" is being used to push through new policies on same-sex education. The hostility is open and raw. But her critiques of the science are as weak and unfounded as she accuses the science to be. Studies of sex differences in neuroanatomy in animals are dismissed as useless because, according to Fine, the field has failed to directly tie differences in the size of the SDN (sexually dimorphic nucleus of the preoptic area, a pronounced sexual dimorphism in the rodent brain) to a specific behavioral change. In this case, the critique is not even accurate, as there is now a growing consensus that the SDN is a key nodal point in the neural process of defeminization and may also be critical to sexual preference. These relatively recent interpretations of SDN function have been appropriately cautious, relying on years of accumulated, converging sources and forms of empirical evidence. Fine's use of Roger Gorski's admission in 1980 [8] that he did not know the functional significance of the nucleus he discovered and her naming of him to further her own arguments smacks of grasping at straws. Moreover, the fact that relatively few neuroanatomical variables have ever been directly tied to the control of behavior seems to have escaped her notice. The use of functional magnetic resonance imaging (fMRI) to detect sex differences in brain activation during specific tasks is rejected by blithely dismissing the entire field of fMRI as too immature and as not capable of really measuring neural activity any way. She has a harder time with the elegant and provocative studies by Melissa Hines [3] and others on sex biases in toy choice in humans and primates, including dismissing studies in CAH girls in which the role of parental influence was carefully assessed and found not to be responsible for these girls' malelike toy preferences [14]. Instead of acknowledging that perhaps there is something interesting going on here, Fine refuses to yield an inch and instead goes through a contorted and ultimately irrational argument about the scientists' "not even knowing" the parameters of male versus female toys that make the toys preferred. Why this undermines the data is unclear. One of her frequent refrains is how little we know, suggesting that this is a flaw of the science as opposed to a natural consequence of the relatively slow pace of discovery resulting from only a small cadre of scientists being focused on and committed to the question at hand. That this situation is complex is not new news, even in the popular press. Melissa Hines's *Brain Gender *[3] is about as balanced a view as possible on the current status of the understanding of both cultural and biological influences on sex differences in brain and behavior. In addition, Anne Fausto-Sterling, author of *Myths of Gender: Biological Theories About Women and Men *[15], relentlessly brings us back to center to remind us how little we really know about the vexingly complex human brain, but she herself shifted from arguing that there is no biological basis for sex differences in the brain to acknowledging there is a complex and as yet poorly understood interaction between biology and society (see *Sexing the Body: Gender, Politics and the Construction of Sexuality *[16]). We fear that books such as the ones by Fine and Jordan-Young will not hasten the pace of discovery, but instead threaten to severely hamper or even reverse the progress that is being made in this field.

Thus, for both authors, endeavors that started as entertaining and potentially enlightening exposés go awry. There is good reason to be irritated with some of what has been written about the neural basis of sex differences in the brain, but the field is a lively and active one in which scientists police each other on a regular basis. There are strong foundations upon which exciting, new and sometimes even startling discoveries are being made. Unilaterally condemning all research on sex differences in the brain because of a few bad players' trying to appeal to a general audience seems akin to claiming that all research on cancer is bogus because some people believe that research suggests cancer can be cured by the power of prayer. In both books, there is a "throwing the baby out with the bathwater" phenomenon in which the entire field is condemned by a combination of setting up straw men to knock down and an overemphasis on criticism of a few errant studies or errant interpretations. What is particularly frustrating to those of us who actively engage in the study of sex differences in the brain is how hard we are continuing to fight for sex or gender as a critical biological variable that must be considered in any study that purports to advance our understanding of the brain. The preference of most neuroscientists is to study only one sex, usually males, and then assume that any findings generalize to females. But time and time again, we have seen this is not the case. This bias is not limited to neuroscience, as a recent analysis demonstrates an overwhelming tendency to exclude females from studies across a wide range of biological disciplines [17]. Moreover, several novel aspects of brain function have been discovered only because males and females were compared, highlighting the tremendous exploratory power of studying sex differences in the brain. These include novel mechanisms of pain regulation, synaptogenesis, neuro- and gliogenesis, cell death, genetic imprinting and surely many more yet to come. Two of the most promising areas for exploitation are (1) gender biases in the relative vulnerability to and the severity of mental health disorders and (2) sex differences in the functional impact of brain damage. By comparing and contrasting the mechanistic basis of sex differences, as well as the therapeutic benefits of various approaches, both sexes stand to benefit. But the useful side of research on sex differences appears to be entirely lost on Fine and, to some extent, on Jordan-Young. *Delusions of Gender *concludes with a depressing final admonition that the cycle of repression of girls is unlikely to change, because it is a self-perpetuating system in which parents teach their children to live along narrow gender lines and those children in turn become parents who then teach the same thing to their children. Fine acknowledges that large numbers of women are taking on hard science and math majors in school, becoming physicians and scientists, but nonetheless she seems to see little hope for change. Nothing short of stopping research on the topic would seem to satisfy her. Jordan-Young, on the other hand, does not go nearly as far as Fine, but rather provides her view of how such research should be conducted and interpreted. Her views again are certainly welcome and valuable. However, she presents them as if they are unknown to scientists studying sex differences. Her observations that "sex" steroids are misnamed because androgens can be converted to estrogens and that these steroids do many other things besides regulating sexuality are not news to those of us in the field who have been inculcated since the 1970 s about the significance of the aromatization hypothesis [18]. Again, such concerns are not put in a broader context. The fact that natural substances are often misnamed and inadequately categorized when they are first discovered is a widespread problem not limited to work on sex steroids. One need only think about what happened during the neuropeptide revolution, when endogenous messengers with names such as vasoactive intestinal polypeptide were found to be widespread and active within the brain. The idea that experience effects interact with hormonal effects in fascinating ways is also not a new observation and has often come out of research trying to discern the context in which hormones act. One cannot help but think that Jordan-Young's focus on human studies, which, as she states, cannot involve detailed experimental analyses, led her to assume an overly deterministic view of hormone action on behavior that is not indicative of the broader field. She points to the value of adopting a broader biological context by considering sex differences as a particular type of reaction norm which addresses the variability in phenotypes that a single genotype can generate in different environments. This is a valuable concept that is in widespread use by researchers, especially evolutionary ecologists, who are trying to understand the causes of intraspecific phenotypic variation. Sex differences are indeed just another example of intraspecific variation, albeit one that is perhaps better demarcated than some other dimensions. An exciting development in the behavioral field is to consider personalities in animals and humans in the context of reaction norms, and it makes sense to include sex as another variable in this context. The challenge, of course, is ascertaining the full response range of a trait specified by a particular reaction norm and testing this response range in a variety of environments. Such a thorough analysis is not going to happen in humans for the obvious reasons mentioned in Jordan-Young's book, though it would be a valuable research strategy to pursue in nonhuman animals. However, it is a bit disappointing to be told that studies in plants and animals are essential to understanding sex differences in a broader biological context when she is prescribing a future research strategy after ignoring or even denigrating such studies (for example, the guinea pigs studied by Phoenix *et al*. [10] are "lowly" in her eyes) when she considers studies of the organizational and activational hypothesis.

But popular books are written to appeal to a broader audience, and in that respect both Jordan-Young and Fine have succeeded. Prompting laypeople to adopt a more critical view of overly simplistic views of complex data sets is a goal any scientist can support, and for that we applaud their efforts. Pendulums tend to swing widely before the inexorable progression to the center. Where that center will ultimately be is the critical issue, and ensuring that it is appropriately balanced is in large part the responsibility of scientists who must continue to do innovative and useful research into the topic of sex differences in brain and behavior.
